# Supplementary material for: Ultrastrong and multifunctional aerogels with hyperconnective network of composite polymeric nanofibers
Source: Nat Commun. 2022 Jul 22;13:4242. doi: 10.1038/s41467-022-31957-2 (PMC9307841; doi:10.1038/s41467-022-31957-2)
Supplement: Supplementary file 1 — Supplementary Information [file 41467_2022_31957_MOESM1_ESM.pdf]

Supplementary Information

Ultrastrong and Multifunctional Aerogels with Hyperconnective Network of Composite Polymeric Nanofibers

Huimin He[1,5], Xi Wei[1,5], Bin Yang[1,2], Hongzhen Liu[1], Mingze Sun[1], Yanran Li[3], Aixin Yan[3], Chuyang Y. Tang[4], Yuan Lin[1,2]✉ and Lizhi Xu[1,2]✉

[1]Department of Mechanical Engineering, The University of Hong Kong, Hong Kong SAR, China.

[2]Advanced Biomedical Instrumentation Centre Limited, Hong Kong SAR, China.

[3]School of Biological Sciences, The University of Hong Kong, Hong Kong SAR, China.

[4]Department of Civil Engineering, The University of Hong Kong, Hong Kong SAR, China.

[5]These authors contributed equally: Huimin He and Xi Wei.

✉e-Email: xulizhi@hku.hk (L.X); ylin@hku.hk (Y.L.)

Supplementary Figures 1-19

Supplementary Tables 1-2

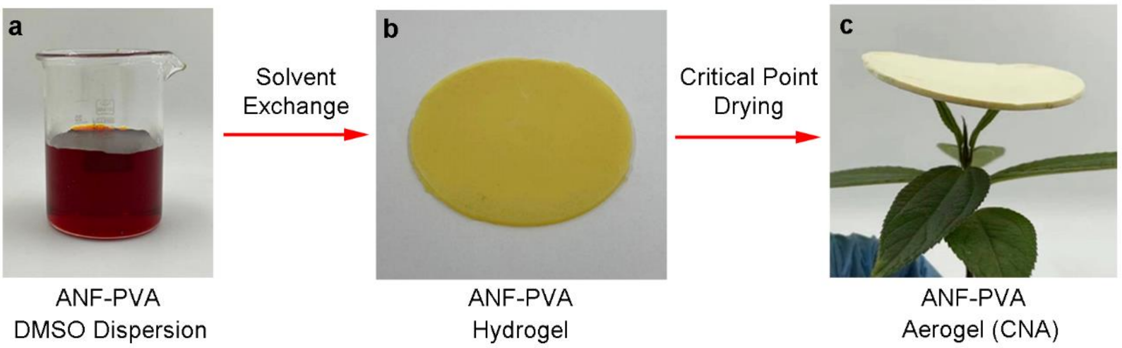

Supplementary Fig. 1 **Photographs of ANF-PVA composites under different states. a,** ANF-PVA dispersion in DMSO. **b,** A hydrogel sample after solvent exchange. **c,** An aerogel sample generated by supercritical CO_2 drying.

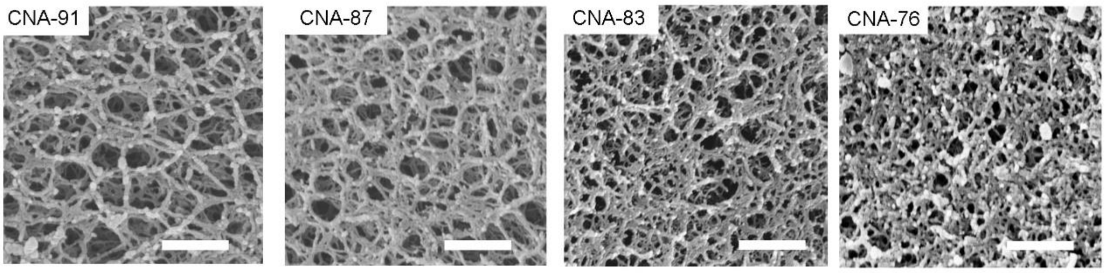

Supplementary Fig. 2 **SEM images of CNAs with various porosity.** Scale bar: 500 nm.

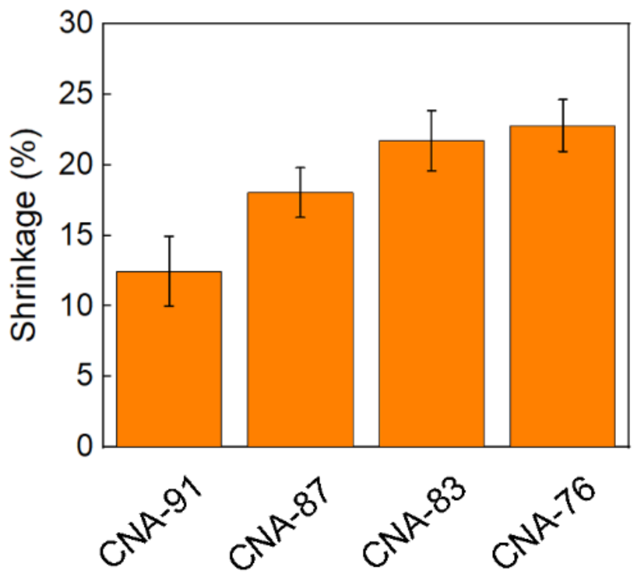

Supplementary Fig. 3 **Shrinkage of CNAs during CPD processes.** The degree of shrinkage increases with increasing solid content.

(**Fig. 2c**).

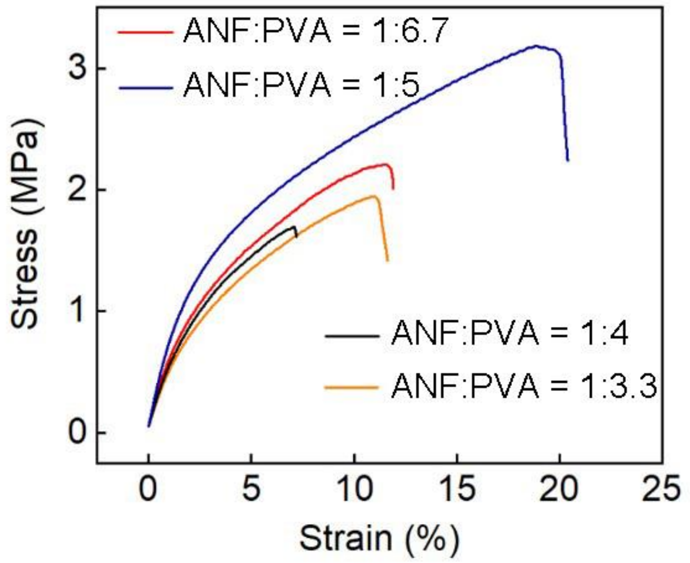

Supplementary Fig. 4 **Effects of the ANF-PVA mixing ratio on the tensile properties of CNAs**. The samples have a fixed porosity of ~83%. An optimized mass ratio of 1:5 between ANF and PVA was identified for both high modulus and strength.

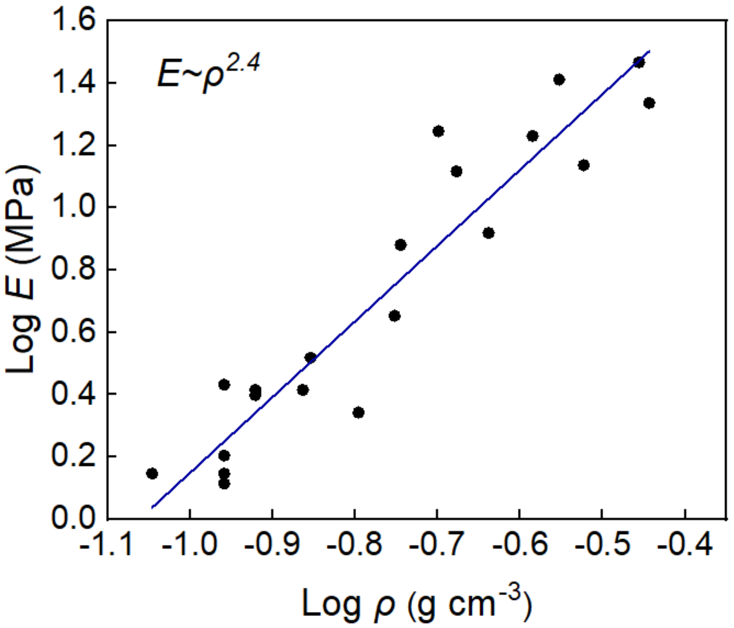

Supplementary Fig. 5 **Compressive modulus of CNAs as a function of density**. This function follows a typical power law for aerogel materials and compares favorably with that for tension (**Fig. 2c**).

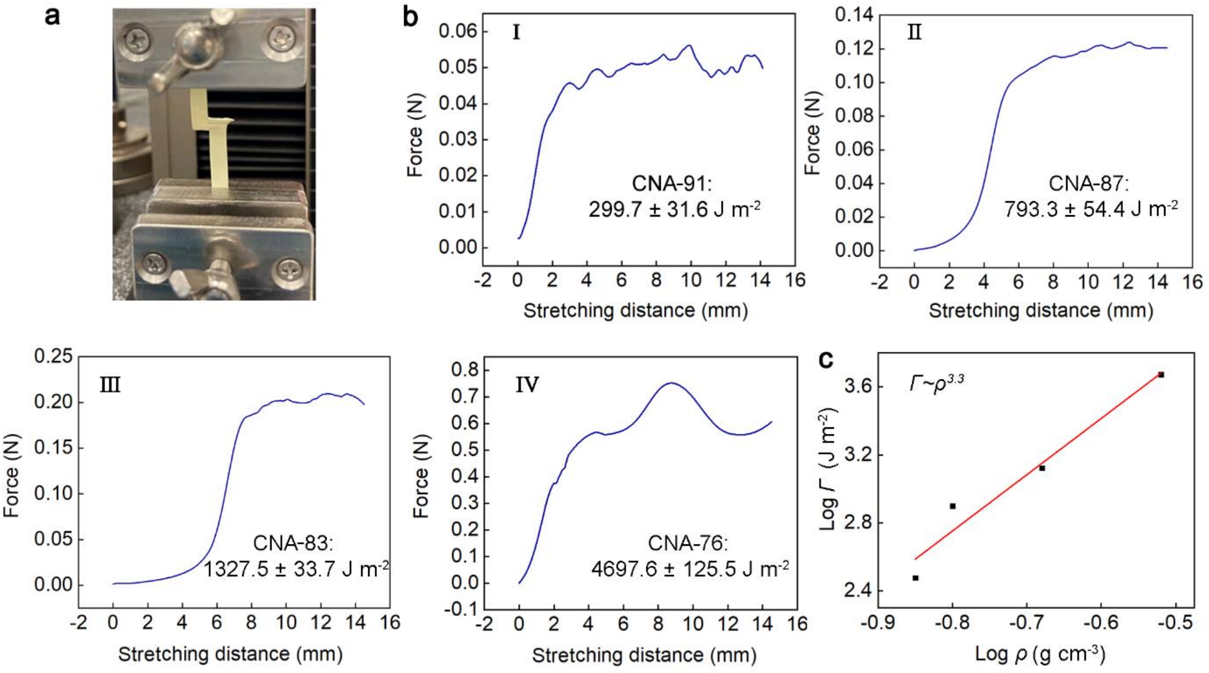

Supplementary Fig. 6 **Fracture energies of CNAs characterized by tearing tests. a,** A photograph of the experimental setup. **b**, Force-extension curves of tearing test and the calculated tearing energies for CNA 91 (I), CNA 87 (II), CNA 83 (III) and CNA 76 (IV). **c,** Logarithmic plot of the fracture energy as a function of density.

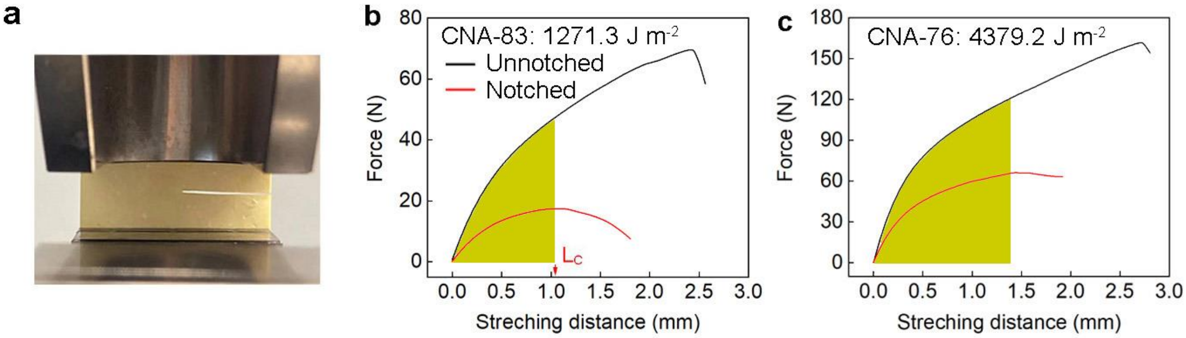

Supplementary Fig. 7 **Fracture energies of CNAs characterized by pure shear tests. a,** A photograph of the experimental setup. **b,c,** Force-extension curves for notched and unnotched samples, as well as the calculated fracture energy for CNA 83 **(b)** and CNA 76 **(c).**

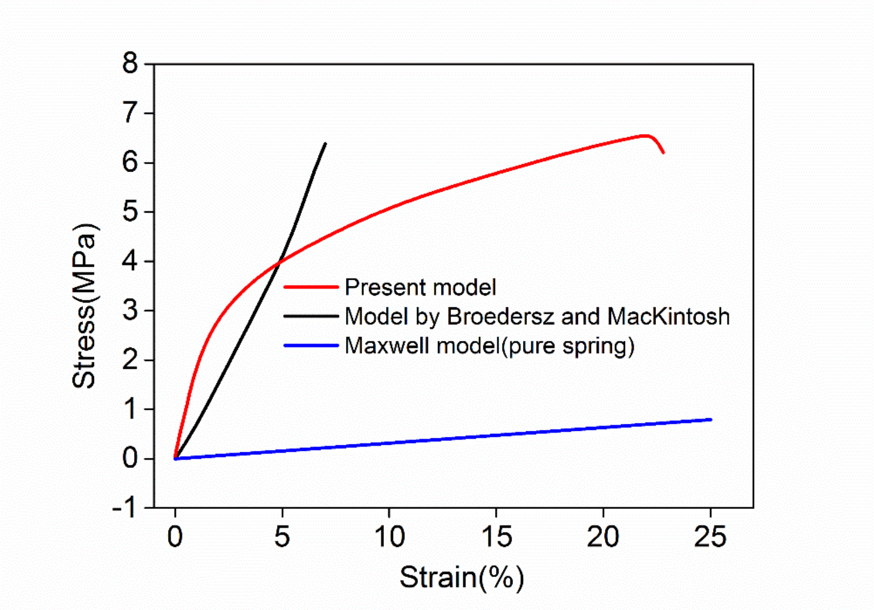

Supplementary Fig. 8 **Predicted tensile stress-strain responses of a fibrillar network (with connectivity $\bar{z}$ =5.4) according to different models.** Each fibril was treated as linear spring (i.e. having negligible bending rigidity) in the Maxwell model[1]. In this case, we can see that the resistance of the network against imposed deformation is very small, which consistent with the fact that the connectivity here is below the threshold level of $z = 6$ (for a network to exhibit non-vanishing bulk modulus) predicted by the Maxwell theory. The bending rigidity of fibrils was taken into account in the model by Broedersz and MacKintosh[2], but connected fibrils were allowed to freely rotate with respect to each other (i.e. fibrils are hinged together). Notice that, in this case, the network exhibits no strain-softening because crosslinker breakage was not considered. The red line corresponds to results from the present model.

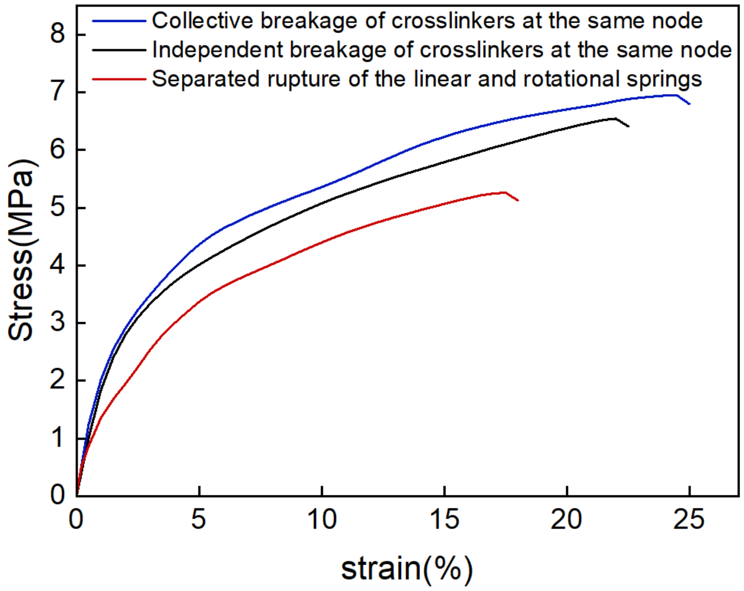

Supplementary Fig. 9 **Stress-strain curves of CNA-76 under different crosslinker failure criteria.** For a welded node containing $\boldsymbol{N_f}$ fibers, there are $\boldsymbol{N_c} = \frac{\boldsymbol{N_f(N_f-1)}}{\boldsymbol{2}}$ crosslinkers interconnecting different fibers. If we assume these $\boldsymbol{N_c}$ crosslinkers fail simultaneously once the total strain energy stored in them reaches $\boldsymbol{N_c E_c}$ (with $\boldsymbol{E_c}$ being the average binding energy of each crosslinker), the simulated tensile response of CNA is shown by the blue line. Clearly, under such circumstance, the stress and failure strain all become slightly higher than those when crosslinkers were allowed to break independently (i.e. when the strain energy stored in any on them is above $\boldsymbol{E_c}$). This is not surprising because some crosslinkers will undergo larger distortion than others and therefore will break first, which weakens the whole material and eventually leads to a lower stress level and fracture strain. On the other hand, if we assume that the linear and rotational springs in each crosslinker can rupture independently (i.e. once the energy stored in them reaches $\boldsymbol{E_c}/2$), then the material will rupture at an even lower stain level (red curve). Further examination of our simulation results revealed that the strain energy stored in the linear spring in most crosslinkers is higher than that in the rotational spring. Consequently, many liner springs will break earlier under this new criterion, ultimately resulting in the earlier fracture of the whole material. Nevertheless, it can be seen that the overall shape/trend of the stress-strain curve under different breakage criteria of crosslinkers remains largely the same, indicating the main physics should have been captured by the model.

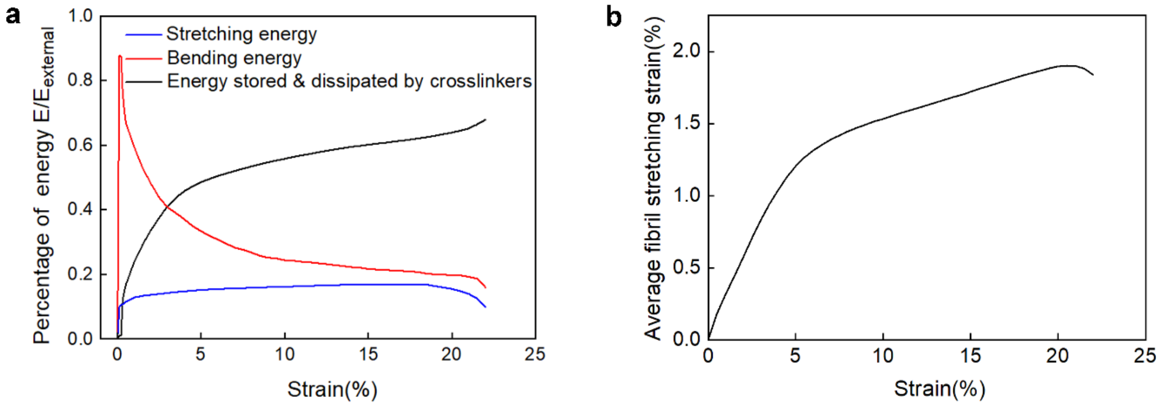

Supplementary Fig. 10 **The role of fibril stretching in the simulated tensile response of CNAs. a,** Comparison of different energies stored and dissipated in the deformed network. **b,** The average stretching strain of fibrils during the deformation process. These results show that the response of CNAs is mostly governed by the bending, enforced deformation and breakage of crosslinkers at nodal points, rather than the stretching and fracture of individual fibrils.

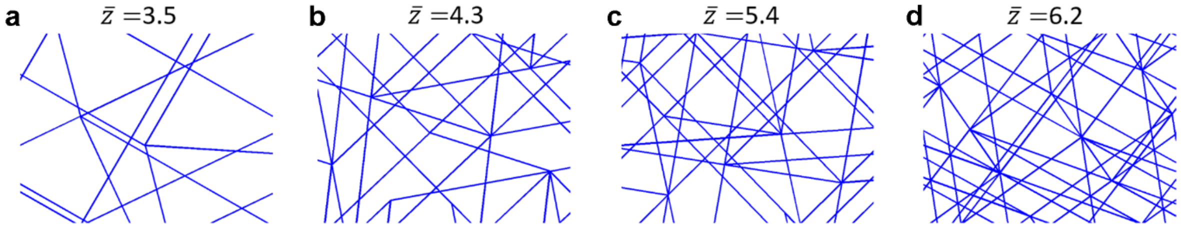

Supplementary Fig. 11 **Representative 3D networks with distinct average nodal connectivity ($\bar{z}$) defined as the average number of fibers connected to each node.** The $\bar{z}$ values were set to be 3.5 **(a)**, 4.3 **(b)**, 5.4 **(c)**, and 6.2 **(d)**, respectively.

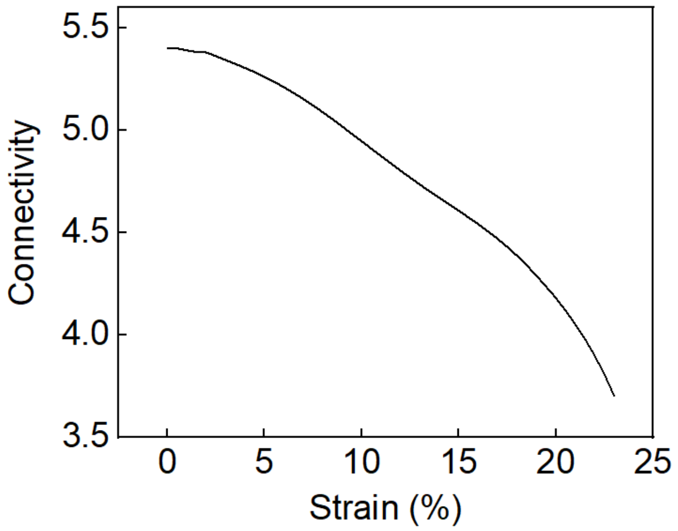

Supplementary Fig． 12 **The average connectivity of the network as a function of the imposed strain.** Not surprisingly, the connectivity decreases monotonically with the strain, reflecting that crosslinkers will break successively as the deformation increases.

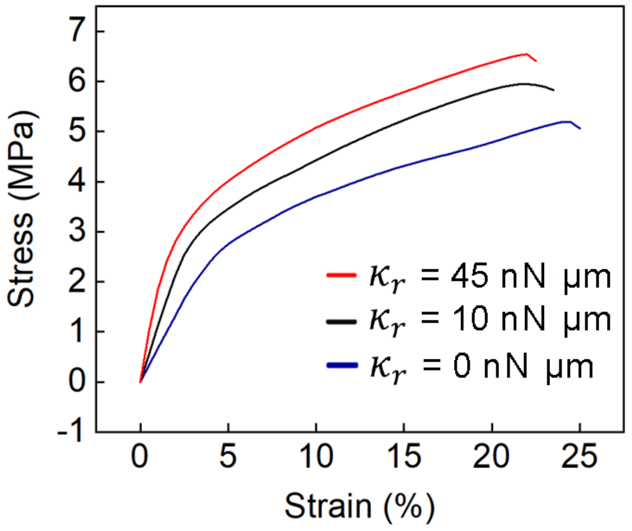

Supplementary Fig．13 **Simulated stress-strain response of CNAs with different rotation stiffness (κ_r) of crosslinkers**. The initial modulus of networks increases with κ_r. In addition, larger rotation stiffness leads to an earlier onset of strain-softening, indicating the crosslinkers begin to break at smaller strain levels under such circumstance.

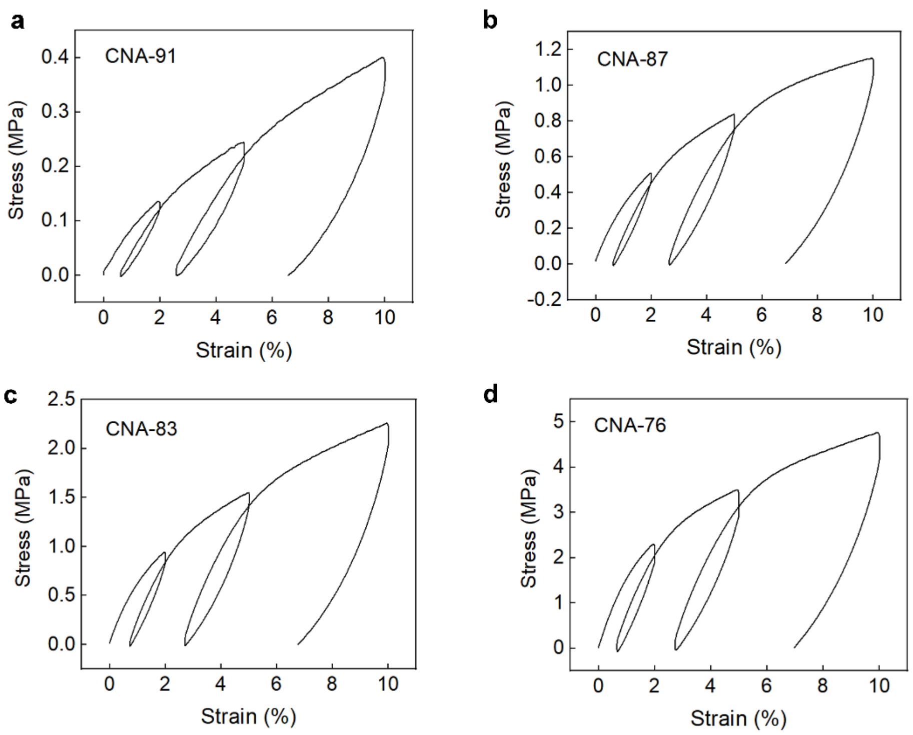

Supplementary Fig.14 **Multiple hysteresis strain cycles on isotropic CNAs.** Response of CNA-91 (**a**), CNA-87 (**b**), CNA-83 (**c**), and CNA-76 (**d**) under three cycles of tensile deformation, with an imposed strain of 2, 5, and 10% respectively.

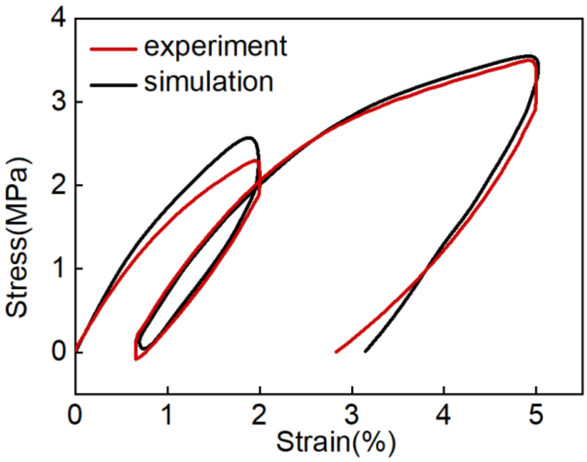

Supplementary Fig. 15 **Comparison between the simulated and measured hysteresis response of CNA-76.** Simulation parameters adopted here are the same as those used in Fig. 3.

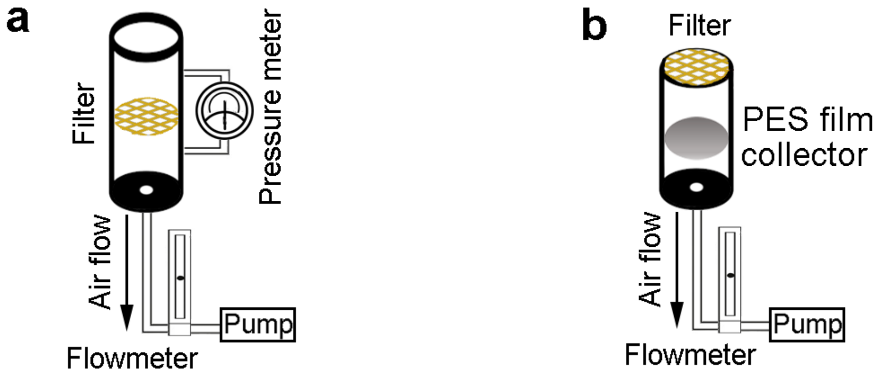

Supplementary Fig. 16 **Schematics of the characterization for air filtration membranes. a,** Air permeability. **b,** Filtration of airborne bacteria.

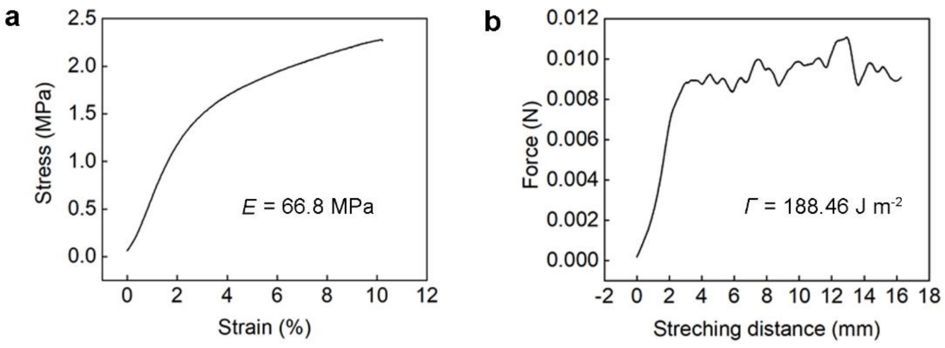

Supplementary Fig. 17 **Mechanical properties of MCE films. a,** Tensile stress-stain curve. **b,** Force-extension curves of tearing test and the calculated tearing energies.

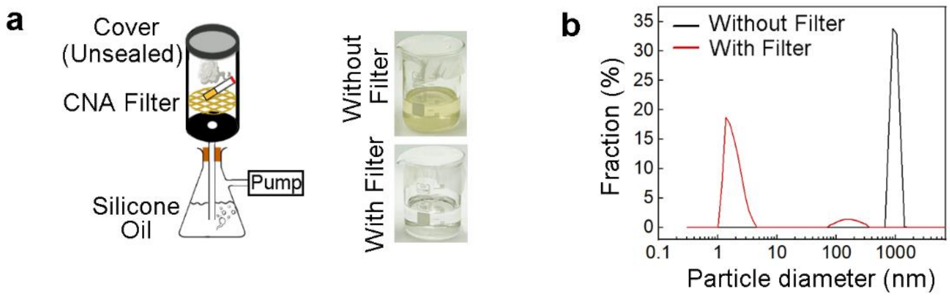

Supplementary Fig. 18 **Filtration of airborne particulate matter by CNA membranes**. **a,** A schematic of the experimental setup. Smoke was filtered by a CNA membrane with the filtered air pumped though silicone oil. Photographs show that the silicon oil is clear with the filtration, as compared with the dark-colored silicone oil without filtration. **b**, Particle size distribution in the silicone oil with or without filtration, showing that the CNA filter effectively removed the majority of the airborne particulate matter with a mean particle diameter of ~ 1 μm.

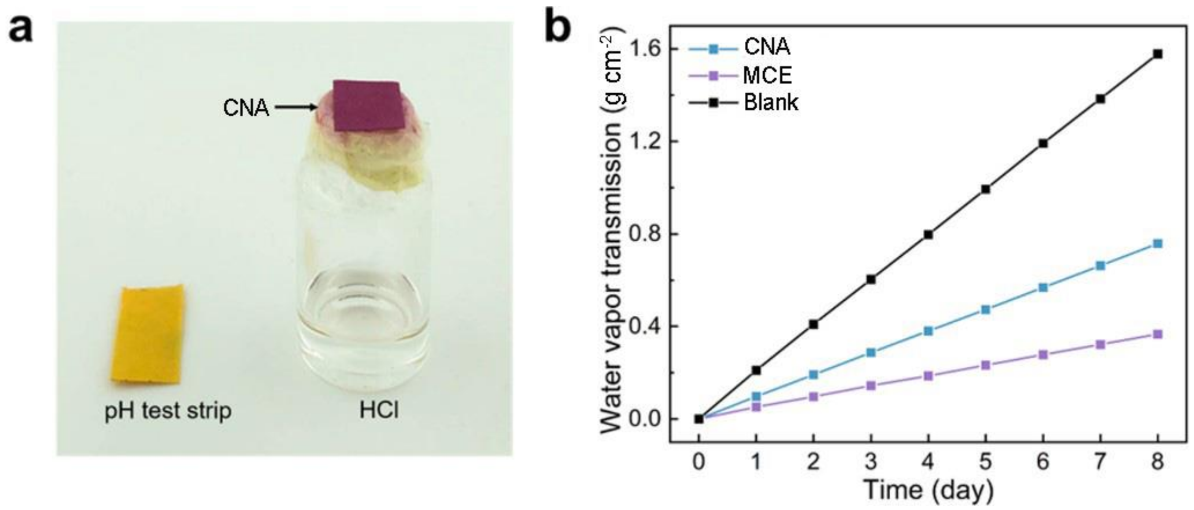

Supplementary Fig. 19 **Vapor permeability of CNA films. a,** A pH test strip turned red on the top of bottle filled with concentrated HCl and sealed with a CNA film. **b,** water vapor permeability of CNA film in comparison with MCE film. The water vapour transmission rate (WVTR) was measured by the gravimetric "cup" method.[3] CNA-83 films with a thickness of 20 μm were used to seal openings of individual test cups filled with DI water and placed in a desiccator cabinet with fixed relative humidity (RH 25%) and temperature (25°C) for 8 days. Commercial MCE films (MF-Millipore™ Membrane Filter, 0.22 μm pore size) were used as controls. Samples were weighed every 24 h to calculate weight loss.

Supplementary Table 1 **Parameters of CNAs as compared with other polymeric aerogels with high mechanical properties.**

	Density ($g\ cm^{-3}$)	Tensile modulus (MPa)	Specific tensile modulus ($MPa\ cm^3\ g^{-1}$)	Toughness ($kJ\ m^{-3}$)	Ref.
Crosslinked Polyimide (PI)	0.3	167.5	558.3	362.3	4
PI/Graphene	0.01	1.7	173.8	9	5
PI/CNT	0.4	59.8	149.5	152.8	6
Bacterial Cellulose (BC)	0.32	14.5	45.2	207.4	7
BC/PEDOT/ SWCNT	0.35	84	240	22.7	8
Chitin	0.17	80.5	473.5	480.7	9
Chitosan	/	/	550.6	147.6	10
CNA-91	0.14	19.1	136.4	212	This work
CNA-87	0.16	32.9	205.6	406	
CNA-83	0.21	67.6	321.9	523	
CNA-76	0.30	187.6	625.3	1050.6	

Supplementary Table 2 **Parameters used in the simulation.**

Connectivity z	Mean distance l_c (μm)	Linear spring stiffness κ_s ($\mu N\ \mu m^{-1}$)	Rotational spring stiffness κ_r (nN μm)	Breakage energy E_m (nN μm)
6.2	0.267	72	45	10
5.4	0.25	72	45	10
4.3	0.22	72	45	10
3.5	0.2	72	45	10

References

1 Maxwell, J. On the calculation of the equilibrium and stiffness of frames. *Mag. J. Sci.* **27**, 294-299 (1864).

2 Broedersz, C. P. & MacKintosh, F. C. Modeling semiflexible polymer networks. *Rev. Mod. Phys.* **86**, 995-1036 (2014).

3 Macedo, I. S. M., Sousa-Gallagher, M. J., Oliveira, J. C. & Byrne, E. P. Quality by design for packaging of granola breakfast product. *Food Control* **29**, 438-443 (2013).

4 Guo, H. Q. *et al.* Tailoring properties of cross-linked polyimide aerogels for better moisture resistance, flexibility, and strength. *ACS Appl. Mater. Interfaces* **4**, 5422-5429 (2012).

5 Qin, Y. Y. *et al.* Lightweight, superelastic, and mechanically flexible graphene/polyimide nanocomposite foam for strain sensor application. *ACS Nano* **9**, 8933-8941 (2015).

6 Liu, P., Tran, T. Q., Fan, Z. & Duong, H. M. Formation mechanisms and morphological effects on multi-properties of carbon nanotube fibers and their polyimide aerogel-coated composites. *Compos. Sci. Technol.* **117**, 114-120 (2015).

7 Wan, J. Q., Zhang, J. M., Yu, J. & Zhang, J. Cellulose aerogel membranes with a tunable nanoporous network as a matrix of gel polymer electrolytes for safer lithium-ion batteries. *ACS Appl. Mater. Interfaces* **9**, 24591-24599 (2017).

8 Jia, F. *et al.* High thermoelectric and flexible pedot/swcnt/bc nanoporous films derived from aerogels. *ACS Sustainable Chem. Eng.* **7**, 12591-12600 (2019).

9 Ding, B. *et al.* Light weight, mechanically strong and biocompatible α-chitin aerogels from different aqueous alkali hydroxide/urea solutions. *Science China Chemistry* **59**, 1405-1414 (2016).

10 Gong, Y. *et al.* Synthesis and characterization of graphene oxide/chitosan composite aerogels with high mechanical performance. *Polymers* **11**, 777 (2019).
